# Reply to the letter to the editor: “What are the effects of diagnostic imaging on clinical outcomes in patients with low back pain presenting for chiropractic care? A matched observational study.” Jenkins et al., Chiropractic & Manual Therapies 2021;29:46

**DOI:** 10.1186/s12998-022-00421-9

**Published:** 2022-03-01

**Authors:** Hazel J. Jenkins, Alice Kongsted, Simon D. French, Tue Secher Jensen, Klaus Doktor, Jan Hartvigsen, Mark Hancock

**Affiliations:** 1 Faculty of Medicine, Health and Human Sciences, Macquarie University, Room 2232, Level 2, 75 Talavera Rd, Sydney, 2109 Australia; 2 Department of Sports Science and Clinical Biomechanics, University of Southern Denmark, Odense, Denmark; 3 Chiropractic Knowledge Hub, Odense, Denmark; 4 Faculty of Medicine, Health and Human Sciences, Macquarie University, Sydney, Australia; 5 Silkeborg Regional Hospital, Silkeborg, Denmark

Reply to the letter to the editor

We thank Dr Lopes for his interest in our study [1] and his recognition of its importance.

We agree that our study did not specifically describe treatment or how the imaging performed may (or may not) have influenced treatment. As we stated in the original article, our aim was to determine whether treatment outcomes (e.g. pain intensity, disability) changed when the chiropractor decided to use imaging as part of patient management, not to explore how imaging may affect the specific treatment provided by chiropractors.

The primary purpose of imaging, as recommended by clinical practice guidelines, is to rule out serious pathology [2]. In our study, the mean number of patients referred for imaging was 24% and up to 64% of patients were referred for imaging depending on the individual chiropractor. Therefore, it is likely that imaging was not being performed to rule out serious pathology alone, which occurs in less than 1% of low back pain presentations [3]. Rather, some chiropractors may have been referring for imaging when they thought it likely to change or inform patient management.

In our study we attempted to approximate randomisation by matching patients on known baseline data, including intention to use spinal manipulation. By doing this we attempted to make the matched groups as similar as possible so that the chiropractor’s decision to refer, or not refer, for imaging would be the key difference between the groups. We also controlled for the chiropractor in the analysis to account for possible differences between individual chiropractors. As described in the limitations section of our article, we could not account for unmeasured variables; however, we considered that key variables likely to affect the decision to refer for imaging were accounted for.

For these reasons we believe that our conclusion that the decision to refer for imaging did not result in better clinical outcomes is appropriate. A randomised controlled trial is indicated to further explore this area without the limitations inherent in a matched observational design.

We also agree with Dr Lopes that future studies to describe how imaging informs treatment and whether different types of imaging assessment change clinical outcomes are needed. We will note though that in the treatment of low back pain, there is currently no evidence that spinal manipulation techniques that use imaging as part of the clinical decision making about where and how to perform the treatment produce superior results [4, 5].

## Data Availability

Not applicable.
